# Chemopreventive effect of resveratrol and apocynin on pancreatic carcinogenesis via modulation of nuclear phosphorylated GSK3β and ERK1/2

**DOI:** 10.18632/oncotarget.5981

**Published:** 2015-10-19

**Authors:** Akihisa Kato, Aya Naiki-Ito, Takahiro Nakazawa, Kazuki Hayashi, Itaru Naitoh, Katsuyuki Miyabe, Shuya Shimizu, Hiromu Kondo, Yuji Nishi, Michihiro Yoshida, Shuichiro Umemura, Yasuki Hori, Toshio Mori, Masahiro Tsutsumi, Toshiya Kuno, Shugo Suzuki, Hiroyuki Kato, Hirotaka Ohara, Takashi Joh, Satoru Takahashi

**Affiliations:** ^1^ Department of Gastroenterology and Metabolism, Nagoya City University Graduate School of Medical Sciences, Nagoya, Japan; ^2^ Department of Experimental Pathology and Tumor Biology, Nagoya City University Graduate School of Medical Sciences, Nagoya, Japan; ^3^ Department of Community-based Medical Education, Nagoya City University Graduate School of Medical Sciences, Nagoya, Japan; ^4^ Radioisotope Research Center, Nara Medical University School of Medicine, Kashihara, Nara, Japan; ^5^ Department of Pathology, Saiseikai Chuwa Hospital, Sakurai, Nara, Japan

**Keywords:** resveratrol, apocynin, pancreatic carcinogenesis, GSK3β, hamster

## Abstract

Despite progress in clinical cancer medicine in multiple fields, the prognosis of pancreatic cancer has remained dismal. Recently, chemopreventive strategies using phytochemicals have gained considerable attention as an alternative in the management of cancer. The present study aimed to evaluate the chemopreventive effects of resveratrol (RV) and apocynin (AC) in *N*-Nitrosobis(2-oxopropyl)amine-induced pancreatic carcinogenesis in hamster. RV- and AC-treated hamsters showed significant reduction in the incidence of pancreatic cancer with a decrease in Ki-67 labeling index in dysplastic lesions. RV and AC suppressed cell proliferation of human and hamster pancreatic cancer cells by inhibiting the G1 phase of the cell cycle with cyclin D1 downregulation and inactivation of AKT-GSK3β and ERK1/2 signaling. Further, decreased levels of GSK3β^Ser9^ and ERK1/2 phosphorylation and cyclin D1 expression in the nuclear fraction were observed in cells treated with RV or AC. Nuclear expression of phosphorylated GSK3β^Ser9^ was also decreased in dysplastic lesions and adenocarcinomas of hamsters treated with RV or AC *in vivo*. These results suggest that RV and AC reduce phosphorylated GSK3β^Ser9^ and ERK1/2 in the nucleus, resulting in inhibition of the AKT-GSK3β and ERK1/2 signaling pathways and cell cycle arrest *in vitro* and *in vivo*. Taken together, the present study indicates that RV and AC have potential as chemopreventive agents for pancreatic cancer.

## INTRODUCTION

Pancreatic cancer is steadily increasing in incidence and has a very poor prognosis [1]. Despite progress in clinical cancer medicine in the fields of imaging technology, surgical management and molecular-targeted therapy, the prognosis of pancreatic cancer has remained dismal. In Japan, about 27,000 patients are estimated to have pancreatic cancer, and almost the same number of deaths are attributable to this cancer annually [2]. Indeed, the 5-year overall survival rate of patients with pancreatic cancer is less than 5% [3]. The dismal prognosis is attributed to aggressive local invasion, early metastasis and low responsiveness to conventional chemotherapies, which indicate that efforts should be directed at developing novel strategies such as chemoprevention to reverse, suppress, prevent or delay the progression of pancreatic cancer [4].

Chemoprevention by naturally-occurring agents in plants, collectively termed phytochemicals, is gaining much attention as a newer dimension in the management of cancer. Many phytochemicals have shown cancer chemopreventive potential in a variety of bioassay systems or animal models which have relevance to human disease. Resveratrol (RV; 3,40,5-trihydroxystilbene) which is one of the promising phytochemicals, is a naturally-occurring polyphenolic compound found in various plants such as grapes and berries and in red wine, and has been found to have broad spectrum beneficial health effects including anti-inflammatory, anti-oxidant and anti-cancer activities [5]. Most of the evidence that shows cancer chemopreventive effects of RV is well documented in various cancers such as those of hepatocellular, lung, skin and prostate by multiple regulatory mechanisms [6–9]. *In vitro* treatment with RV induced cellular apoptosis [10] and inhibited proliferation [11] in human pancreatic cancer cell lines, and significantly suppressed tumor growth and enhanced the therapeutic effect of gemcitabine *in vivo* [12]. However, the chemopreventive effects of RV on early stage pancreatic carcinogenesis using an animal model have not been reported.

Apocynin (AC; 4′-hydroxy-3′-methoxyacetophenone or acetovanillone) is another potential anti-cancer naturally-occurring compound. This phytochemical was identified as the biologically active substance in the roots of *Picrorhiza kurroa* Royle ex Benth, a perennial plant growing in the Himalayan alpines. Extracts from the roots are used in the traditional Ayurvedic practice of India and Sri Lanka for the preparation of ethnic medicines for the treatment of liver, heart, joint and lung ailments [13]. AC disrupts the assembly of the NADPH oxidase complex, which is the enzyme responsible for the production of reactive oxygen species, therefore, inhibition of this enzyme represents an attractive therapeutic target for the treatment of many diseases such as those stated above. Recently, several studies have reported that AC is also able to inhibit tumor cell migration of breast cancer [14], and suppress progression and carcinogenesis of prostate cancer [15, 16]. However, the chemopreventive effects of AC on pancreatic cancer has not been established yet.

In the present study, RV and AC were used to study the potential of these phytochemicals in the chemoprevention of pancreatic cancer using the hamster animal model. The *N*-Nitrosobis(2-oxopropyl)amine (BOP)-treated Syrian golden hamster model exhibits many morphologic and molecular features of human pancreatic cancer progression and has commonly been used as an *in vivo* model in pancreatic cancer studies. Recent studies have used the hamster model for chemopreventive purposes in cancer using drug regimens and for imaging purposes to detect early pancreatic cancer [17–19]. In addition, several studies using this hamster model indicated that a high fat diet promotes pancreatic carcinogenesis [20, 21]. Therefore, we investigated the chemopreventive effects of RV and AC on pancreatic cancer in BOP-treated hamsters under the high fat diet condition. Further, we used RV and AC as promising phytochemicals in chemoprevention of pancreatic cancer, since they are known to induce endogenous adiponectin [22, 23]. A recent epidemiological study has shown that low plasma adiponectin levels are associated with an elevated risk of pancreatic cancer [24], therefore, this study also aimed to elucidate the effects of adiponectin on pancreatic carcinogenesis.

## RESULTS

### Treatments with resveratrol and apocynin have no effect on clinical signs and levels of serum lipids

All animals remained healthy throughout the experimental period. There was no significant difference in the mean body, liver, kidney and visceral fat weights among the groups at the end of the study (Table 1). During the experiment, no significant difference was found in water consumption among the groups. Histologically, there was no toxic change in the liver and kidneys with administration of RV or AC (data not shown). Blood glucose levels and the serum levels of triglyceride, total cholesterol, LDL cholesterol, HDL cholesterol, free fatty acid and amylase are listed in Supplementary Table S1. Blood glucose, total cholesterol and LDL cholesterol levels were mildly decreased in both RV and AC groups as compared to the control group. However, these trends were not significant among the groups. Serum levels of adiponectin were not altered by treatments with RV and AC (Supplementary Figure S1: control, RV and AC: 26.7 ± 2.2, 24.9 ± 2.5 and 25.3 ± 2.8 ng/mL, respectively).

**Table 1:** The body, liver, kidney and visceral fat weights and water consumption of the hamsters at 18-weeks-old

Group	Number of hamsters	Treatment	Body weight(g)	Liver weight(g)	Kidney weight(g)	Visceral fat weight(g)	Water consumption (ml /day)
Control	13	BOP	185.0 ± 24.1	10.7 ± 1.9	1.2 ± 0.2	4.3 ± 1.4	9.1 ± 1.5
RV	12	BOP + RV	187.9 ± 16.8	10.9 ± 1.4	1.2 ± 0.3	4.1 ± 1.2	10.0 ± 1.2
AC	12	BOP + AC	185.3 ± 15.2	10.7 ± 1.2	1.2 ± 0.2	4.0 ± 0.8	9.3 ± 1.5

RV, Resveratrol; AC, Apocynin

### Resveratrol and apocynin inhibit progression of pancreatic tumorigenesis as well as cell proliferation in BOP-treated hamsters

**Figure 1:**
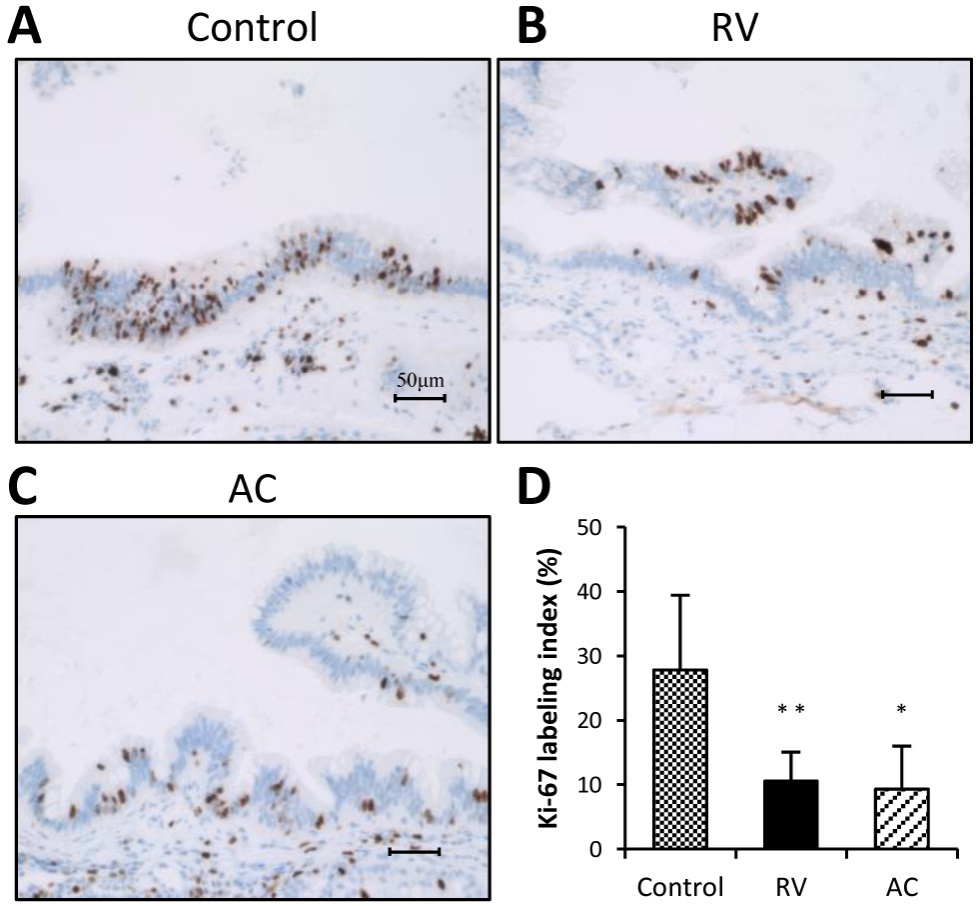
Ki-67 immunohistochemistry and labeling indices of pancreatic dysplasia in BOP-treated hamsters **A–C.** Representative features of Ki-67 immunohistochemical staining in the control group (A), RV treatment group (B), and AC group (C) Bars = 50 μm. RV, resveratrol; AC, apocynin. **D.** RV and AC significantly decreased the Ki-67 labeling index in dysplastic lesions as compared to the control group (*P < 0.05 and **P < 0.01).

As summarized in Table 2, dysplasia and ductal adenocarcinoma developed in the pancreas of all groups, and there was no difference in the incidence of dysplasia between the groups. However, the incidence of adenocarcinomas in both RV and AC groups was significantly decreased as compared to those in the control group (control, RV and AC: 54%, 8% and 8%, respectively, *p* < 0.05). The proliferative potential of pancreatic ductal dysplasia was investigated by Ki-67 immunostaining (Figure 1). The Ki-67 labeling index in ductal dysplasia was significantly decreased by RV and AC treatments (*p* < 0.01 and *p* < 0.05, respectively).

**Table 2:** The effects of RV and AC on the incidence of neoplastic lesions in the pancreas

Group	No. of animals with lesions (%)	
	dysplasia	adenocarcinoma
Control (13)	5 (38)	7 (54)
RV (12)	5 (42)	1 (8)*
AC (12)	5 (42)	1 (8)*

RV, Resveratrol; AC, Apocynin

* Significantly different from control group (*P* < 0.05).

### Resveratrol and apocynin inhibit pancreatic cancer cell proliferation and induce G1 phase cell cycle arrest

**Figure 2:**
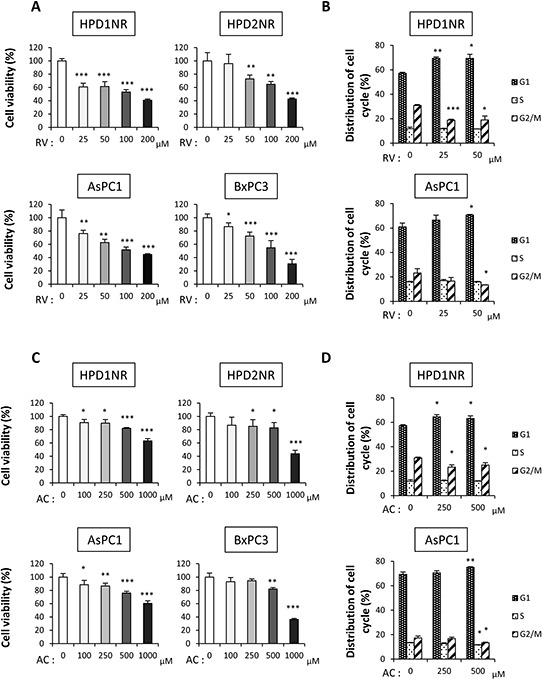
Anti-proliferation effects of RV and AC in hamster (HPD1NR and HPD2NR) and human (AsPC1 and BxPC3) pancreatic cancer cell lines **A.** and **C.** Significant decreases in the cell viability of four cell lines were observed in a dose-dependent manner after RV (A) and AC (C) treatments by the WST-8 assay. Values are means ± SD, *n* = 4. **B.** and **D.** Guava^®^ cell cycle analysis of HPD1NR and AsPC1 treated with RV (B) or AC (D) Values are means ± SD, *n* = 3 in each, *P < 0.05, **P < 0.01 and ***P < 0.001 as compared to controls.

To elucidate the mechanisms of anti-carcinogenesis by RV and AC, we further explored the functional role of RV and AC in cell proliferation of hamster (HPD1NR and HPD2NR) and human (AsPC1 and BxPC3) pancreatic cancer cell lines. RV and AC significantly inhibited the growth of four cell lines in a dose-dependent manner (Figure 2A and 2C). Combined with the reduction of cell proliferation by RV and AC *in vivo*, these *in vitro* observations led us to examine whether RV and AC modulate cell cycle progression in pancreatic cancer cells. RV- and AC-treated HPD1NR and AsPC1 cells appeared to accumulate in the G1 phase as compared to the controls, with concomitant decrease in the percentage of cells in the G2/M phase (Figure 2B and 2D).

### Resveratrol and apocynin decrease phosphorylation of AKT and ERK1/2 and reduce cyclin D1 expression

**Figure 3:**
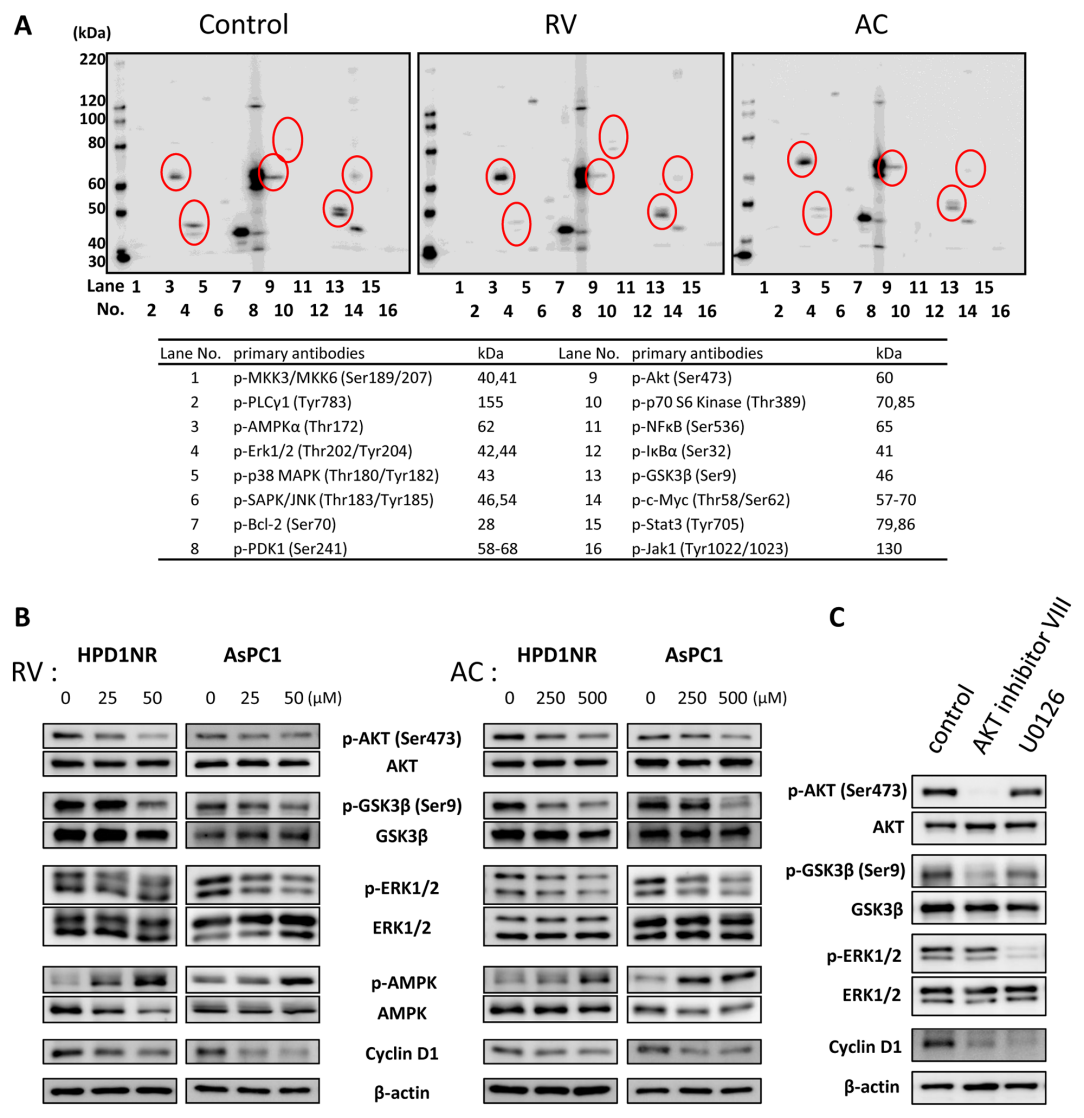
Regulation of AKT/GSK3β and ERK1/2 signaling pathways by RV and AC treatments **A.** Western blot analysis of multiple phosphorylated proteins associated with cell proliferation in AsPC1 cells. The lane numbers are for the primary antibodies on the right side. Significant spots are indicated with open circles. **B.** Immunoblot analysis of whole cell lysates of HPD1NR and AsPC1 cells. RV and AC inhibit AKT, GSK3β^Ser9^ and ERK1/2 phosphorylation in a dose-dependent manner. The results demonstrated are representative of three independent experiments. **C.** AsPC1 cells were incubated for 24 hours with specific inhibitors of AKT (AKT inhibitor VIII, 10 μM) and ERK1/2 (U0126, 10 μM) or without inhibitor (Control), and analyzed by Western blotting for expression of cyclin D1.

To understand the potential molecular mechanisms underlying the inhibition of cell cycle by RV and AC in pancreatic cancer cells, we analyzed the signaling pathways associated with cell proliferation using AsPC1 cells. As illustrated in Figure 3A, RV suppressed the levels of phospho-ERK1/2, phospho-AKT (Ser473), phospho-GSK3β (Ser9) and phospho-c-Myc (Thr58/Ser62). Moreover, phospho-AMPKα and phospho-p70 S6 Kinase were highly expressed in RV-treated cells as compared with the controls. Similar results were obtained with AC-treated cells except for the expression pattern of phospho-p70 S6 Kinase.

To further investigate the differential regulation of these signaling pathways by RV and AC in hamster and human pancreatic cancer cell lines, we performed Western blot analysis (Figure 3B). Treatments with RV and AC reduced the levels of phosphorylated ERK, AKT and GSK3β^Ser9^ in a dose-dependent manner, whereas they promoted the level of phospho-AMPKα. However, no change was shown at the Tyr216-phosphorylation site of GSK3β in AsPC1 cells (Supplementary Figure S2). In addition, we observed an effect of RV on Thr58 and Ser62 phosphorylation using separate and cocktail antibodies; phosphorylation at both sites were decreased by RV treatment. However, since total c-Myc expression was also decreased, the ratios of Thr58- and Ser62-phosphorylated c-Myc to total protein were not altered in RV-treated cells. On the other hand, AC did not reduce Thr58/Ser62-phosphorylated and total c-Myc, and this was not reproducible result as compared to multiple phosphorylated protein analysis (Supplementary Figure S3).

At the same time, the expression of cyclin D1 was dramatically down-regulated in both RV- and AC-treated cells. In order to investigate the regulation of cyclin D1 expression in pancreatic cancer cells, we used specific inhibitors of the signal transduction pathways. AsPC1 cells were incubated with inhibitors of AKT (AKT inhibitor VIII, 10 μM) and ERK1/2 (U0126, 10 μM) for 24 hours and analyzed by Western blotting for expression of cyclin D1. As shown in Figure 3C, a decrease in cyclin D1 expression was observed in the AsPC1 cells treated with AKT and ERK1/2 inhibitors, suggesting the involvement of these pathways in the up-regulation of cyclin D1 in pancreatic cancer cells.

### Inhibition of cell proliferation is due to reduction of nucleus-localized cyclin D1 expression through the pathway involving nuclear GSK3β and ERK1/2

**Figure 4:**
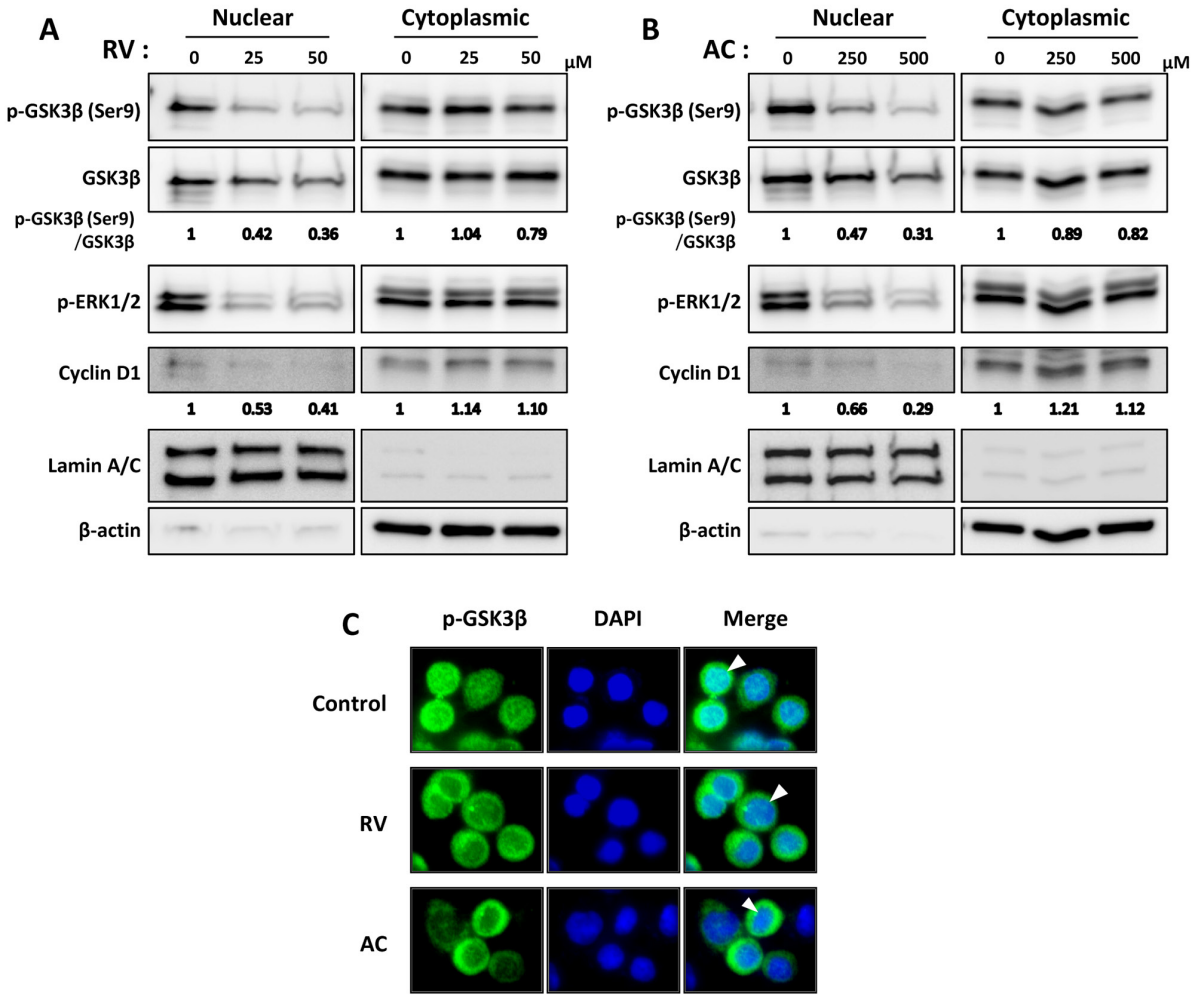
Involvement of nuclear phosphorylated GSK3β^Ser9^ and ERK1/2 in anti-proliferation effects of RV- and AC-treated pancreatic cancer cells **A.** and **B.** Western blot analysis of nuclear and cytoplasmic fractions shows decreased nuclear phosphorylated GSK3β^Ser9^ and ERK1/2, and cyclin D1 expression after RV (A) and AC (B) treatments. Lamin A/C and β-actin antibodies were used to confirm nuclear and cytoplasmic fractions, respectively. The results demonstrated are representative of three independent experiments. Values below blots indicate relative band intensities determined as described above, justifying the use of Lamin A/C or β-actin levels for normalization of the nuclear or cytoplasmic cyclin D1 expression, respectively. **C.** Immunocytochemical analysis of phosphorylated GSK3β^Ser9^ localization. Control and RV- or AC-treated cells were immunostained with anti-phosphorylated GSK3β^Ser9^ antibody (green), and the nuclei were visualized with DAPI (blue). Arrowhead : staining showing nuclear localization of phosphorylated GSK3β^Ser9^.

Next, to verify the subcellular localizations of phosphorylated GSK3β^Ser9^ and ERK1/2, we further examined protein expression of nuclear and cytoplasmic fractions in human pancreatic cancer AsPC1 cells by Western blotting (Figure 4A and 4B). Phosphorylated GSK3β^Ser9^ and ERK1/2 were observed in both the nucleus and cytoplasm of AsPC1, and those in the nucleus were decreased by both RV and AC treatments in a dose-dependent manner. Further, the expression of cyclin D1 in the nuclear fraction was reduced, but not in the cytoplasmic fraction. Immunocytochemical staining also confirmed decreased phosphorylated GSK3β^Ser9^ in the nucleus in RV- and AC-treated cells (Figure 4C).

### Phosphorylated GSK3β^Ser9^ expression in the nucleus of ductal dysplasia is reduced by RV and AC

**Figure 5:**
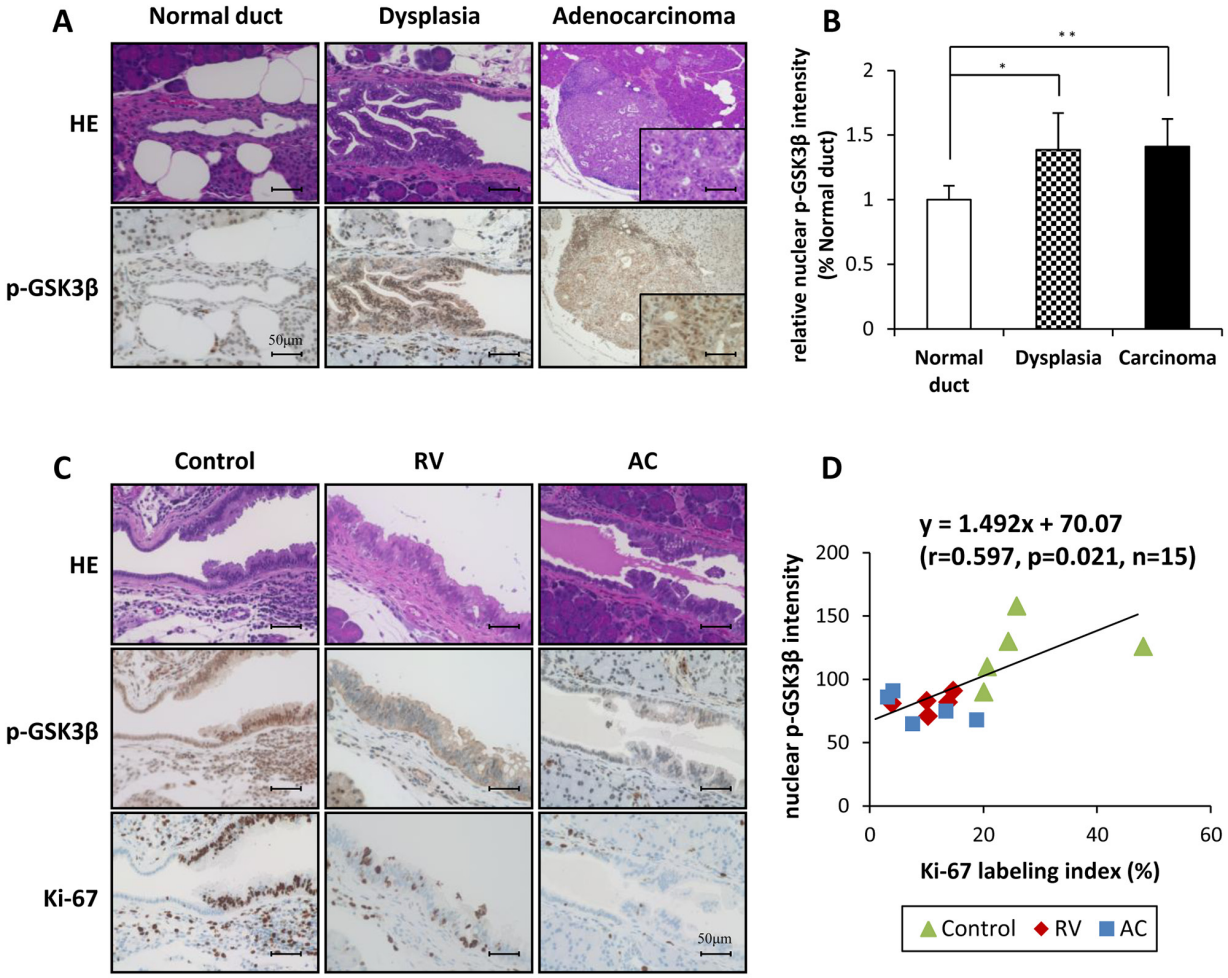
Representative histopathological appearance and immunohistochemical analysis of phosphorylated GSK3β^Ser9^ in the pancreas of BOP-treated hamster **A.** and **B.** Comparison of staining for nuclear phosphorylated GSK3β^Ser9^ between normal duct, dysplasia and adenocarcinoma of the control group of BOP-treated hamsters. Bars = 50 μm. The intensity score of nuclear phosphorylated GSK3β^Ser9^ in each group, *P < 0.05 and **P < 0.01 as compared to normal duct. **C.** and **D.** The staining for nuclear phosphorylated GSK3β^Ser9^ in dysplasia of RV- and AC-treated hamsters was lower than that in the control group. Calculation of the regression line based on the relationship between nuclear phosphorylated GSK3β^Ser9^ and Ki-67 labeling index by Pearson's correlation (*r* = 0.597, *p* = 0.021, *n* = 15).

We next analyzed the expression and localization of these proteins in hamster pancreatic tissue. As illustrated in Figure 5A, phosphorylated GSK3β^Ser9^ was mainly localized in the nucleus of dysplastic and adenocarcinoma tissues, and the intensity in both lesions was significantly higher than that in normal duct of control hamsters (Figure 5B). The level of phosphorylated GSK3β^Ser9^ in the nucleus of dysplastic lesions tended to be lower in RV- and AC-treated groups than that in the control group (Figure 5C). In addition, the amount of phosphorylated GSK3β^Ser9^ was correlated with the Ki-67 labeling index in dysplastic lesions in each group (Figure 5D). These immunohistochemical data suggest that RV and AC also control cell proliferation via modulation of GSK3β activity during hamster pancreatic carcinogenesis.

**Figure 6:**
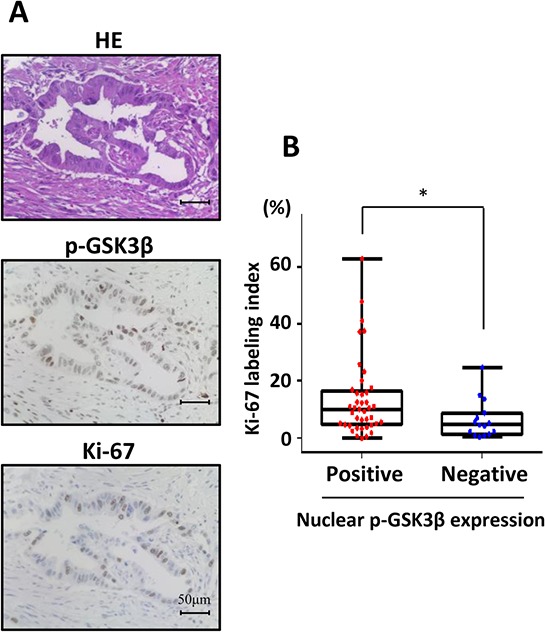
Analysis of proliferative activity using human pancreatectomy specimens according to positive or negative nuclear phosphorylated GSK3β^Ser9^ expression **A.** Representative immunohistochemical staining for phosphorylated GSK3β^Ser9^ in human pancreatic cancer. **B.** Correlation analysis of Ki-67 labeling index and nuclear phosphorylated GSK3β^Ser9^ expression in human pancreatic cancer (*P < 0.05).

To further verify that phosphorylated GSK3β^Ser9^ expression in the nucleus is related to human pancreatic cancer, we analyzed the expression pattern of phosphorylated GSK3β^Ser9^ in pancreatectomy specimens (Figure 6A). We divided them into two groups according to presence or absence of phosphorylated GSK3β^Ser9^ in the tumors. The clinical characteristics of all entry patients are listed in Supplementary Table S3. In the analysis of 56 pancreatic cancer patients, 41 patients (73.2%) were positive for nuclear phosphorylated GSK3β^Ser9^. Moreover, a significant correlation was found between the expression of phosphorylated GSK3β^Ser9^ in the nucleus and Ki-67 labeling index in these samples (Figure 6B). These results suggest that nuclear phosphorylated GSK3β^Ser9^ has an important role in the adaptation of pancreatic cancer to growth.

## DISCUSSION

Pancreatic cancer has a poor prognosis and low survival rates for patients stricken with this disease. Despite improved treatment modalities during the last decade, there has been no substantial improvement in overall survival. Consequently, there is an urgent need for the development of novel therapies for this devastating disease. Recently, phytochemicals, which are compounds from natural sources, have received considerable attention as cancer chemopreventive agents. However, there has not been any report regarding the effectiveness of RV and AC on the prevention of pancreatic carcinogenesis. The present study demonstrated suppressive effects of RV and AC on pancreatic carcinogenesis through regulation of cell proliferation without any adverse effects in an *in vivo* hamster model.

It has been reported that RV and AC induce endogenous adiponectin [22, 23], which is an anti-diabetic and anti-inflammatory adipokine, and its plasma concentration is decreased in obesity [25]. Many case control and prospective studies have shown that the serum concentration of adiponectin is decreased in breast, liver and colorectal cancers [26]. One prospective study reported that low prediagnostic levels of circulating adiponectin were associated with an elevated risk of pancreatic cancer [24]. These observations suggest that adiponectin has a protective role against cancer. Therefore, along with our other goal, we simultaneously investigated whether or not adiponectin inhibits pancreatic carcinogenesis. In contrast to our expectations, analyses using ELISA and RT-PCR revealed that serum concentrations of adiponectin did not differ among all groups in the present *in vivo* study (Supplementary Figure S1). Therefore, we could not clarify the correlation between adiponectin and pancreatic cancer. These findings suggest that RV and AC act as a brake on pancreatic carcinogenesis in an adiponectin-independent manner.

RV has anti-tumor effects against various cancers, including pancreatic cancer. Roy *et al* reported that RV targets the Forkhead box O transcription factors through the inhibition of PI3K/AKT and MEK/ERK signaling, and induces growth arrest and apoptosis in pancreatic cancer cells [27]. Another study indicated that RV inhibits proliferation and induces apoptosis in pancreatic cancer cells by targeting the hedgehog pathway [28]. These findings are consistent with our *in vitro* study which showed that RV induced growth inhibition and G1 arrest in pancreatic cancer cells. Furthermore, a similar effect was also observed in ductal dysplasia and adenocarcinoma in RV-treated hamsters. On the other hand, RV did not induce apoptosis in pancreatic carcinogenesis *in vivo* according to TUNEL assay (data not shown), although RV induced apoptosis in pancreatic cancer cells *in vitro* (Supplementary Table S2). In addition, it is known that the increase in cell proliferation can account for carcinogenicity [29]. Hence, our results suggested that inhibition of cell proliferation may be an essential mechanism to prevent pancreatic carcinogenesis by RV.

There is a lack of data that demonstrates the mechanism by which AC inhibits cell proliferation of various cancers. Moreover, no previous study has revealed an association between AC and pancreatic cancer. With regard to the mechanisms of anti-tumor effects by AC in pancreatic cancer, interestingly, we detected that AC reduced the phosphorylation of AKT, GSK3β^Ser9^ and ERK1/2 which was also observed with RV treatment. Further studies are needed to identify the molecular mechanisms involved in more upstream target signaling. These common pathways may contribute to cyclin D1 degradation because GSK3β and ERK1/2 are critical regulators of cyclin D1 expression [30, 31].

GSK3β has various roles in cancer which even after years of study remain complex and controversial [32]. Several reports showed that GSK3β is overexpressed in various tumor types including pancreatic cancer [33]. In addition, a previous study indicated that GSK3β is a potential therapeutic target for pancreatic cancer [34]. However, the evaluation of GSK3β inhibitors in clinical trials has been hampered by the fear that inhibition of GSK3β may stimulate malignant transformation [35]. In the Wnt signaling pathway, active GSK3β phosphorylates β-catenin at serine 33, which primes β-catenin for ubiquitination and subsequent proteasome-mediated degradation. Inhibition of GSK3β leads to the stabilization of β-catenin, resulting in nuclear translocation of β-catenin and promotion of transcription of β-catenin target genes including cyclin D1 [36]. Although recent evidence suggests that Wnt/β-catenin signaling may contribute to pancreatic carcinogenesis [37], in the present study, we did not find any significant differences in the phosphorylation level of cytoplasmic GSK3β and subsequent nuclear β-catenin by both RV and AC treatments (data not shown). Another study indicated that GSK3β has a nuclear localization sequence [38], and has also been shown to play an important role in the determination of cyclin D1 expression level by directly regulating transcription and protein degradation [30]. In addition, a previous study reported that nuclear GSK3β promotes nuclear export and proteolysis of cyclin D1, resulting in cell cycle arrest in the G1 phase [39]. In concordance with previous findings, our results showed that nuclear localization of phosphorylated GSK3β^Ser9^ and subsequent cyclin D1 expression were decreased by both RV and AC treatments. Likewise, it has also been reported that GSK3β is required for phosphorylation of c-Myc [40]. Our results indicated that RV reduced c-Myc as well as cyclin D1 expression resulting in suppression of cell proliferation, while the phosphorylation of c-Myc was not affected by both agents. At the same time, the connection between GSK3β and K-ras is still obscure, although it is important to clarify this connection since K-ras mutations occur in more than 90% of pancreatic cancers. A recent study has indicated that mutant K-ras-mediated signaling increases GSK3β expression in pancreatic cancer cells by the MAPK signaling pathway [41]. On the other hand, a previous study on colon cancer cells reported that the K-ras oncoprotein transactivates β-catenin via inactivation of GSK3β [42]. However, this effect was dependent on PI3K, but did not appear to require inhibitory phosphorylation at Ser9.

Our results illustrated that the ERK as well as the GSK3β pathways are related with the inhibition of proliferation through the regulation of cyclin D1. It is known that nucleocytoplasmic shuttling is an important determinant of ERK function. In quiescent cells, ERK is predominantly localized in the cytoplasm, and once fully phosphorylated by MEK, ERK is released from cytoplasmic scaffold proteins and rapidly translocates to the nucleus [43, 44]. ERK1/2 activation has been associated with pancreatic tumorigenesis [45], and ERK promotes cyclin D1 transcription and assembly with CDK subunits [39, 46]. On the other hand, another study indicated that activated ERK1/2 upregulates cyclin D1 expression through inactivation of GSK3β by phosphorylation at Ser9 [47]. These differing findings propelled us to further investigate the mechanistic details of this pathway.

Collectively, our results clearly demonstrate that RV and AC down-regulate phosphorylation of AKT, GSK3β^Ser9^ and ERK1/2, suppress cell proliferation, and inhibit pancreatic carcinogenesis, without any signs of tissue toxicity. Further, our findings provide new information on possible mechanisms of action for RV and AC in pancreatic cancer cells which involve regulation of the subcellular localization of GSK3β and ERK1/2. Thus, RV and AC warrant further attention as promising chemopreventive agents for pancreatic cancer.

## MATERIALS AND METHODS

### Animals, diet and chemicals

Five-week-old female Syrian golden hamsters weighing approximately 80 g were purchased from Japan SLC, Inc. (Shizuoka, Japan) and acclimated to the laboratory for one week. They were housed three animals per cage on pulp-chip bedding in an air-conditioned animal room at 22 ± 2°C and 55 ± 5% humidity. All hamsters were maintained under specific pathogen-free conditions with a 12-h light/dark cycle. The Quick Fat diet (crude fat, 13.6%; crude protein, 24.2%; total calories, 4.06 kcal/g) (CLEA Japan, Tokyo) was used as a high fat regimen. RV and AC were purchased from Sigma-Aldrich (St. Louis, MO) and BOP was obtained from Toronto Research Chemicals Inc. (Toronto, Canada).

### Animal treatment and biochemical analysis

A total of thirty-seven female hamsters at 6 weeks of age received four subcutaneous injections of BOP (on days 1, 3, 5 and 7) at a dose of 10 mg/kg body weight. One week after the last BOP injection, they were randomly divided into three groups, and all groups were given a high fat diet. The RV and AC groups were subsequently given drinking water containing 200 mg/L RV or 500 mg/L AC, respectively, for 10 weeks, and the control group was provided water without any reagent. Food and water were available *ad libitum*, and their consumption along with body weight were measured weekly. At the end of the experiment when the hamsters were 18-weeks-old, all hamsters were anesthetized, and blood samples were collected from the aorta. Visceral fat was assessed by weighing total adipose tissues surrounding the uteri after dissection. Blood glucose levels were measured using an automatic blood glucose meter (Medisafe-mini GR-102; Terumo, Tokyo, Japan). Serum were stored at −80°C until being assayed for triglyceride, total cholesterol, LDL cholesterol, HDL cholesterol, free fatty acid and amylase using radioimmunoassays by the Tohkai Cytopathology Institute : Cancer Research and Prevention (TCI-CaRP, Gifu, Japan). Adiponectin concentrations in serum were determined by ELISA (R&D Systems, Inc, Minneapolis, MN). All animal experiments were performed under protocols approved by the Institutional Animal Care and Use Committee of Nagoya City University Graduate School of Medical Sciences.

### Histopathological examination

At necropsy, the pancreas, kidneys, liver and bile duct were carefully examined macroscopically. Three anatomical parts of the pancreas (the gastric, splenic and duodenal lobes) were fixed in 10% phosphate-buffered formalin after spreading on filter paper or frozen for RNA extraction. All tissues were routinely processed, embedded in paraffin, serially sectioned to 4 μm thick, and stained with hematoxylin and eosin (H&E) to assess the histopathological features. Pancreatic lesions were histopathologically diagnosed as dysplasia or adenocarcinoma. Dysplasia corresponds to lesions in human that are referred to as PanIN1, 2 and 3. To assess cell proliferation in dysplasia, deparaffinized sections were treated with a Ki-67 antibody (Abcam, Cambridge, MA, 1:100 dilution), and the proliferating cells were quantified by counting the Ki-67-positive cells at a magnification of × 400.

### Cell culture

Human pancreatic cancer cells, AsPC1 and BxPC3, were obtained from the American Type Culture Collection (ATCC, Rockville, MD), and maintained in RPMI1640 (Wako Pure Chemical Industries Co. Ltd., Osaka, Japan) supplemented with 10% fetal bovine serum (FBS). Hamster pancreatic cancer cells, HPD1NR and HPD2NR [48], were maintained in DMEM (Wako Pure Chemical Industries Co. Ltd.) supplemented with 10% FBS. Cells were cultured at 37°C in 5% CO_2_ humidified air. Cell authentication (STR profile) of all human cancer cell lines (AsPC1 and BxPC3) was performed by JCRB cell bank on May 11, 2015.

### Cell viability assay

Cell viability was analyzed by the WST-8 cell proliferation assay. AsPC1, BxPC3, HPD1NR and HPD2NR cells were seeded into 96-well culture plates at a concentration of 1.0 × 10^4^ cells/200 μL/well and incubated overnight. Cells were subsequently treated with RV (0–200 μM) or AC (0–1000 μM). After incubation for 48 hours, cells were incubated for 2 hours with the Cell Counting Kit-8 (Dojindo, Kumamoto, Japan) according to the manufacturer's protocol, and the absorption at 450 nm was measured with a microplate spectrophotometer (SPECTRA MAX340; Molecular Devices, Kenilworth, NJ). Cell viability was expressed as a percentage of untreated control cells.

### Cell cycle analysis

AsPC1 and HPD1NR cells were treated with RV or AC for 24 hours, then suspensions were prepared and stained with propidium iodide (Guava Cell Cycle Reagent, Guava Technologies, Hayward, CA) according to the Guava Cell Cycle Assay protocol. Cell cycle phase distributions were determined on a Guava PCA Instrument using CytoSoft Software.

### Western blotting

AsPC1 and HPD1NR cells were cultured with RV or AC for 24 hours, and we used AKT inhibitor VIII and U0126 (Calbiochem) as specific inhibitors. Cell monolayers were rinsed thrice with ice-cold PBS (Sigma) and prepared according to the extraction method. To prepare whole cell samples, cells were dissolved in 100 μL of cell lysis buffer (Cell Signaling Technology, Lake Placid, NY). For nuclear and cytoplasmic extracts, trypsinized cells were washed in PBS, resuspended in cell lysis buffer of the Nuclear/Cytosol Fractionation Kit (BioVision, Inc., Mountain View, CA) and then treated according to the manufacturer's protocol. Protein concentrations were determined with a Protein Assay Kit (Bio-Rad Laboratories, Hercules, CA). Aliquots of samples were fractionated by 8–16% sodium dodecyl sulfate-polyacrylamide gel electrophoresis and then electroblotted onto nitrocellulose membranes. The membranes were then incubated with the following antibodies: phospho-MKK3/MKK6, phospho-PLCγ1, phospho-AMPKα, AMPKα, phospho-Erk1/2, Erk1/2, phospho-p38MAPK, phospho-SAPK/JNK, phospho-Bcl-2, phospho-PDK1, phospho-Akt (Ser473), Akt, phospho-p70 S6 Kinase, phospho-NFκB, phospho-IκBα, phospho-GSK3β (Ser9), GSK3β, phospho-c-Myc (Thr58/Ser62), c-Myc, phospho-Stat3, phospho-Jak1, cyclin D1, Lamin A/C (Cell Signaling Technology), phospho-GSK3β (Tyr216), phospho-c-Myc (Thr58), phospho-c-Myc (Ser62) and β-actin (Abcam). Immunoreactions were demonstrated by the ECL-Plus detection system (GE Healthcare, Piscataway, NJ) after 1 hour incubation with secondary horseradish peroxidase-labeled anti-rabbit or anti-mouse antibody (Cell Signaling Technology). The Western blot signals were quantitated by densitometric analysis using ImageQuant TL (GE Healthcare).

### Clinical patients and pancreatic cancer specimens

We used the computerized database of the institution to identify 56 patients with pancreatic cancer who underwent surgery between June 1997 and December 2009 at the Department of Gastroenterological Surgery, Nagoya City University Hospital. The surgical specimens were fixed in neutral-buffered 10% formalin, embedded in paraffin, and processed for histopathologic diagnosis and immunohistochemical examinations. This study was approved by the institutional review board at Nagoya City University Hospital.

### Immunohistochemical analysis of phosphorylated GSK3β

For immunohistochemical analysis, deparaffinized sections of hamster and human pancreatic tissues were incubated with 1:100 diluted anti-phospho-GSK3β^Ser9^ antibody (Cell Signaling Technology). Antibody binding was visualized by a conventional immunostaining method using an autoimmunostaining apparatus (HX System, Ventana, Tucson, AZ). The intensity score of nuclear phosphorylated GSK3β^Ser9^ in each lesion was evaluated using the Biorevo BZ-9000 microscope and the associated software (KEYENCE, Osaka, Japan).

### Statistical analysis

Differences in quantitative data, expressed as mean ± SD, between groups were compared by one-way ANOVA, Dunnett's post-hoc test, Mann-Whitney *U* test and the χ^2^ test as appropriate using Graph Pad Prism 5 (GraphPad Software, Inc., La Jolla, CA).

## SUPPLEMENTARY FIGURES AND TABLES


