# Supplementary material for: Chemopreventive effect of resveratrol and apocynin on pancreatic carcinogenesis via modulation of nuclear phosphorylated GSK3β and ERK1/2
Source: Oncotarget. 2015 Oct 19;6(40):42963–75. doi: 10.18632/oncotarget.5981 (PMC4767484; doi:10.18632/oncotarget.5981)
Supplement: Supplementary file 1 [file oncotarget-06-42963-s001.pdf]

SUPPLEMENTARY FIGURES AND TABLES

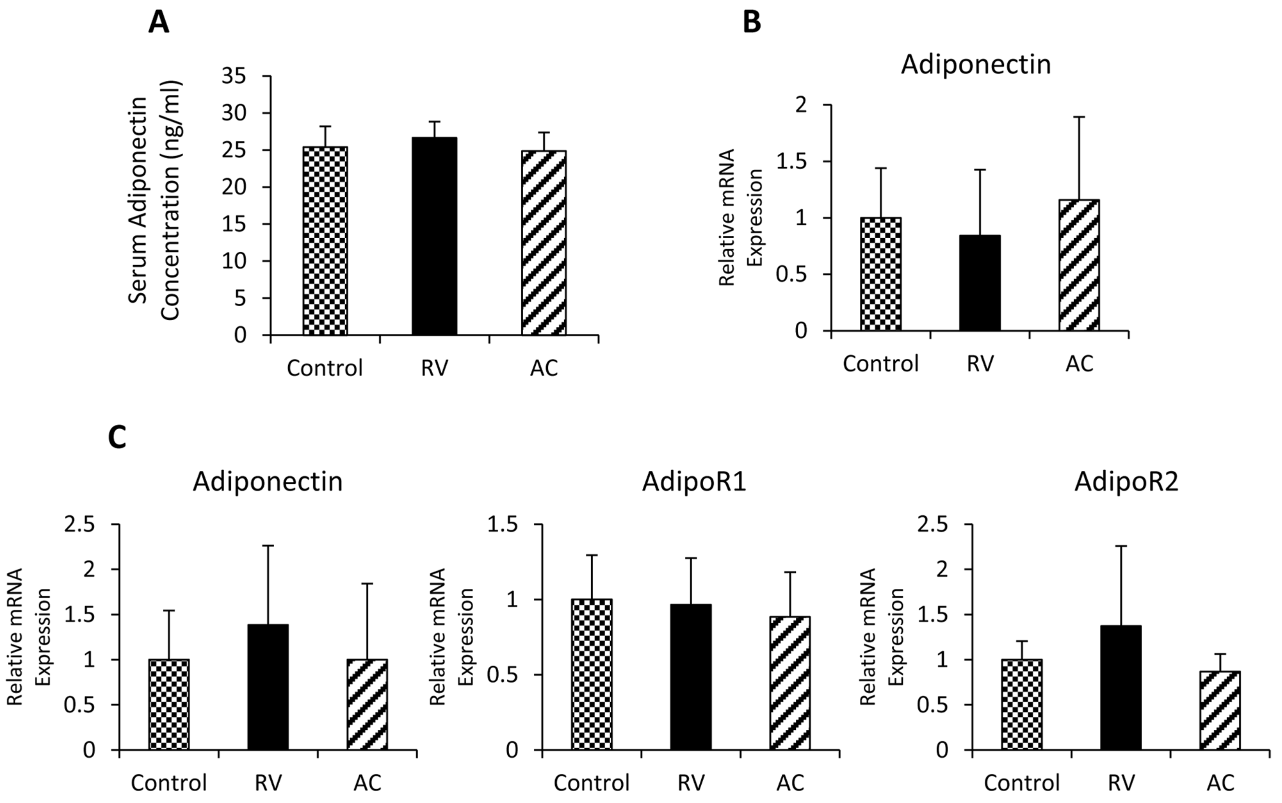

Supplementary Figure S1: The level of endogenous adiponectin in hamsters treated with RV and AC. A. Serum adiponectin (R&D Systems, Minneapolis, MN) was examined using enzyme-linked immunosorbent assay kits according to the manufacture's instructions. **B.** and **C.** Expression analyses of the genes encoding for adiponectin in the visceral fat tissue (B), and adiponectin, adipoR1 and adipoR2 in the pancreatic tissue (C) of hamsters were conducted by quantitative RT-PCR. The amount of mRNA was normalized and shown relative to that of cyclophilin mRNA in each sample.

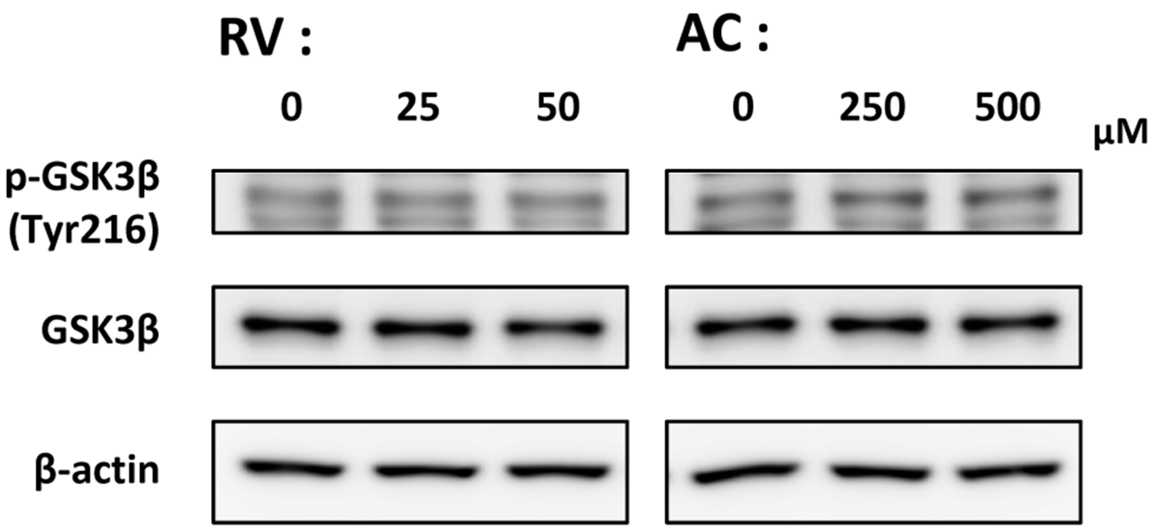

Supplementary Figure S2: Western blot analysis for phosphorylated GSK3β^{Tyr216} in RV- and AC-treated AsPC1 cells. RV and AC did not affect Tyr216-phosphorylated GSK3β.

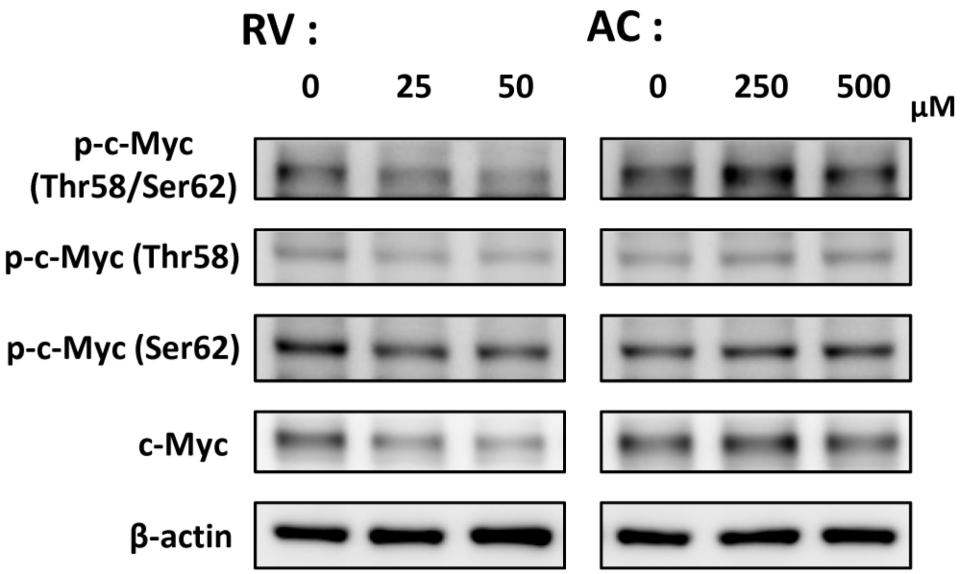

Supplementary Figure S3: The expression of phosphorylated c-Myc in RV- and AC-treated AsPC1 cells. Western blot analysis using total c-Myc and single and cocktail antibodies for Thr58- and Ser62-phosphorylated c-Myc. The results demonstrated are representative of three independent experiments.

Supplementary Table S1: The blood glucose levels and the serum levels of triglyceride, total cholesterol, LDL cholesterol, HDL cholesterol, free fatty acid and amylase in hamsters

Group	Blood glucose (mg/dl)	Triglyceride (mg/dl)	Total cholesterol (mg/dl)	LDL cholesterol (mg/dl)	HDL cholesterol (mg/dl)	Free fatty acid (mEq/l)	Amylase (U/l)
Control (13)	142.0 ± 52.5	483.8 ± 148.4	300.2 ± 49.8	98.3 ± 39.6	144.8 ± 17.8	3.3 ± 0.6	3718.8 ± 515.0
RV (12)	129.3 ± 25.2	476.8 ± 90.8	277.1 ± 26.6	90.2 ± 17.6	136.7 ± 19.7	3.7 ± 0.4	3838.5 ± 686.3
AC (12)	121.9 ± 35.7	524.4 ± 133.2	274.8 ± 24.6	75.3 ± 11.7	144.0 ± 12.3	3.8 ± 0.6	3480.8 ± 500.4

RV, Resveratrol; AC, Apocynin

Supplementary Table S2: The sub-G1 cell population in AsPC1 cells treated with RV or AC

	RV (μM)			AC (μM)		
	0	25	50	0	250	500
Sub-G1 phase (%)	13.3 ± 1.2	22.5 ± 3.8 *	21.4 ± 3.9 *	10.8 ± 2.8	12.0 ± 2.3	12.1 ± 0.5

RV, Resveratrol; AC, Apocynin
Values are means ± SD from three independent experiments
*$P < 0.05$ as compared to controls.

Supplementary Table S3: Patients' characteristics

	Nuclear p-GSK3β expression		*P* value
	Positive (*n* = 41)	Negative (*n* = 15)	
Gender			
Male/female	29 / 12	9 / 6	0.567
Age			
Median (range)	67.4 (32–84)	65.9 (48–79)	0.635
Differentiation			
Well	18	8	0.819
moderate	20	6	
Poorly	3	1	
Stage			
IA, IB, IIA, IIB	29	7	0.177
III, IV	12	8	
T			
1, 2	7	2	0.138
3, 4	33	13	
N			
0	20	5	0.527
1	21	10	
M			
0	38	13	0.489
1	3	2	
